# Double Burden of Rural Migration in Canada? Considering the Social Determinants of Health Related to Immigrant Settlement Outside the Cosmopolis

**DOI:** 10.3390/ijerph16050678

**Published:** 2019-02-26

**Authors:** Asiya Patel, Jennifer Dean, Sara Edge, Kathi Wilson, Effat Ghassemi

**Affiliations:** 1 School of Planning, University of Waterloo, Waterloo, ON N2L 3G1, Canada; 2 Department of Geography & Environmental Studies, Ryerson University, Toronto, ON M5B 2K3, Canada; sedge@ryerson.ca; 3 Department of Geography, University of Toronto Mississauga, Mississauga, ON L5L 1C6, Canada; kathi.wilson@utoronto.ca; 4 Newcomer Centre of Peel, Mississauga, ON L5B 2N6, Canada; eghassemi@ncpeel.ca

**Keywords:** social determinants of health, immigration, rural settlement, health inequality, social inclusion, health care services, housing, employment, gender

## Abstract

There is a large and growing body of research acknowledging the existence of health disparities between foreign-born and native-born populations in many high immigrant-receiving countries. Significant attention has been paid to the role of physical and social environments in the changing health status of immigrants over time. However, very limited attention has been given to these issues within the context of rural geographies, despite global evidence that immigrants are increasingly settling outside of traditional gateway cities and into rural communities. This paper presents the results of a scoping review aimed at assessing the state of knowledge on the health impacts of immigrant migration into rural communities in Canada. Guided by Arksey and O’Malley’s scoping protocol, we conduct a review of academic literature in Canada related to rural migration. A total of 25 articles met inclusion criteria which included access to the social determinants of health. Findings identified a paucity of research directly connecting rural settlement to health but the literature did emphasize five distinct social determinants of health for rural residing immigrants: social inclusion, culturally-appropriate services, gender, employment, and housing. This paper concludes with an identification of research gaps and opportunities for future research into whether rural-residing immigrants face a double burden with respect to health inequity.

## 1. Introduction

There is a large and growing body of research acknowledging the existence of health disparities between foreign-born and native-born populations in many high immigrant receiving countries including Australia [1,2,3,4], the United Kingdom [2] and the United States [2,5]. In Canada, for example, the initially-strong health status of immigrants declines with time spent in the country [6,7], with long-term immigrants reporting lower self-reported health [8,9], higher prevalence of chronic diseases [10] and poorer access to health care services [11,12,13] compared to the Canadian-born population. Health geographers and planners alike have focused on the role of local environments in determining population health broadly, and immigrant health in particular [14,15,16,17]. However, very limited attention has been given to these issues within the context of rural geographies.

In the Canadian context, over 300,000 immigrants arrive annually [18]. Further, foreign-born individuals constitute 22% of the total population, which is the highest proportion in over a century [18]. Over the past 50 years, the settlement location of newcomers has been primarily urban, with 2016 estimates indicating that 91% of Canada’s immigrant population resides in census metropolitan areas (compared to 63% of Canadian-born population) [19]. In fact, over two-thirds of immigrants have settled in Toronto, Montreal, and Vancouver [19]. However, more recent trends indicate that newcomers are settling outside of the core areas of traditional gateway cities in favour of the suburbs [20,21,22,23] and, more recently, small and rural towns [24,25,26,27]. These latter settlement patterns are driven by new policy and planning initiatives, such as Ontario’s Community Immigrant Retention in Rural Ontario [28], the Rural Employment Initiative [29], and federally the Provincial Nominee Program [30], which seek to attract and retain immigrants in aging and declining rural areas. Given the projected and encouraged growth of immigrants to Canada, up to 450,000 newcomers per year by 2025 [31], it is anticipated that rural immigration programs will be a central component of future settlement strategies in Canada.

This “new paradigm” of immigrant settlement outside of traditional gateway cities and into smaller towns and rural areas is an international phenomenon also observed in Australia, New Zealand and the United States [32,33]. Indeed, geographic distribution is mutually beneficial as smaller cities and rural areas need to fill labour shortages and offset population decline, while immigrants benefit from greater employment opportunities and lower costs of living in these areas [25,34]. On the other hand, there is often a lack of physical infrastructure, social service provision, and ethno-cultural resources in rural areas [35,36], factors that are integral to the settlement and well-being of immigrants [24,27]. As part of their rural settlement program, the Canadian government uses a “Welcoming Communities” framework to assess the community readiness of rural areas and small towns seeking to attract newcomers (The term newcomer is often used interchangeably with the term recent immigrant to signify a resident born outside of the country. The former is considered more inclusive and welcoming than the term immigrant and the two terms are used interchangeably in this paper). This framework acknowledges that communities that offer the necessary services, resources and support systems to foster integration and inclusion are more likely to retain immigrants over the long term [27,33,34,37]. These emerging initiatives and the subsequent increase of rural settlement among immigrant populations raises important questions about the health impacts of these changing settlement patterns for newcomer populations and whether rural living exacerbates geographical and health inequities for this population. Specifically, our review paper explores the current state of knowledge on the determinants of immigrant health in rural communities. We have rooted this study in the social determinants of health framework, which acknowledges the pathways through which place affects health, and recognizes geography as a determinant of health [38,39].

### Health Inequities among Immigrant and Rural Populations

Ample research in Canada [4,27,40] and abroad [2,41] has found evidence of a “Healthy Immigrant Effect”, which acknowledges that upon arrival, immigrants are healthier than the general population. Over time, however, the health of immigrants declines to levels at par with or below the Canadian-born population [42]. Several explanatory hypotheses have been presented for this decline, including adoption of unhealthy lifestyles [15,42], poor access to the health care system [11,40,43] which is often exacerbated by discrimination [15], and increased stress associated with the settlement process [11,44,45]. For example, a recent study found that recent immigrants are 1.35 times more likely to meet physical activity guidelines through active commuting (>150 min per week) than the Canadian-born population [46]. The study further found that active commuting declined over time, with recent immigrant 1.78 times more likely to actively commute (>150 min/week) than long-term immigrants [46]. With respect to access to health care, recent immigrants in Ontario, Canada are three times more likely to face barriers accessing specialist care than the Canadian-born population, which largely persists with time spent in the country [47]. A better understanding of the social disparities among this growing portion of the population has been the focus of attention among researchers and policy-makers in Canada over the past 15 years [26,27,48,49,50], yet fewer studies have specifically considered the significance of different geographical contexts within which immigrant health and health care access is experienced.

In studies of non-immigrant populations, health disparities have been found to exist across the rural–urban continuum [36,51,52,53,54]. A national study of rural health in Canada found a positive correlation between all-cause mortality and level of remoteness (outside of metropolitan areas), and that some variation existed across health outcomes and genders [55]. For example, mortality due to circulatory disease is higher for adults in rural areas compared to those in urban areas but mortality due to cancer was lower in rural areas [56]. Rates of injury and road-accident deaths increased with level of remoteness, as did suicide rates, particularly among men [56]. Among adolescents, level of remoteness is positively correlated with obesity in both sexes [57], heavy drinking prevalence in boys, and smoking in girls [55]. Further, research on health care access found that rural residents have poorer access to both primary [12,13] and specialized [47,58] health care services. Men in rural Canada have a life expectancy 2.2 years shorter than their urban counterparts [13].

To date, there is very little research that explores the health of skilled immigrants specific to rural residency. There is work on temporary migrant workers [59], but this population is vastly different from skilled immigrants who intend to reside in Canada permanently. Existing health studies in Canada among long-term immigrants tend to control for rural–urban residence given that very few immigrants reside in rural communities [31]. For instance, in their study of myocardial infarction, new immigrants were less likely than long-term immigrants to have a heart attack, and the authors controlled for rurality, acknowledging that few immigrants reside in rural areas [17].

The significant latency period between social determinants of health and prevalent health outcomes such as diabetes, cancer and cardio-vascular disease makes the assessment of the impacts of changing settlement patterns a challenge. However, research does acknowledge that immigration is itself an important determinant of health [9,44,45,60,61], with regional variations in health being attributed to migration and visible minority status of the population [62]. Consequently, there is an increasing call in the disciplines of geography, planning and public health to be proactive about the long-term health of residents by focusing on access to the social determinants of health in place [14,15,16,28,59,63,64,65]. This paper presents the results of a scoping review to examine the existing evidence base on access to the determinants of health for rural-residing immigrants in Canada. Given the importance of context to understanding the place effects on health [38], we have narrowed the scope to the Canadian context and where political efforts to change the settlement patterns of newcomers is underway.

## 2. Methods

For this review paper, we used a scoping review protocol to search and synthesize relevant bodies of literature in order to map the existing knowledge base on rural immigration and health [66]. In contrast to traditional systematic reviews, scoping studies do not predominantly focus on the strength of association between variables/phenomenon, but rather aim to assess the existing knowledge base and areas of consensus and debate [66]. Scoping reviews have been widely used in health research, particularly for exploratory or emerging work [15,67,68]. Given the paucity of research explicitly focused on immigrant health in rural areas, the use of scoping review methods was desirable in order to assess the existing knowledge base on the social determinants of immigrant health in rural areas. This study was guided by the six-step methodology proposed by Levac et al. (2010) [67], adapted from Arksey and O’Malley’s foundational scoping study guidelines (2005) [66]. Our application of these steps is detailed in Table 1 below.

Given the paucity of research directly examining immigrant health, we searched the literature broadly for all works on rural immigrant settlement in Canada and screened for studies that dealt with health directly and/or focused on social determinants of health. We adopt the comprehensive definition of “health” from the World Health Organization (WHO), which conceptualizes health as “a state of complete physical, mental and social well-being and not merely the absence of disease or infirmity” [33]. We also used the WHO’s (2011) definition of Social Determinants of Health to aid the analysis of results:

*“Societal conditions in which people are born, grow, live, work and age … [including] early years’ experiences, education, economic status, employment and decent work, housing and environment, and effective systems of preventing and treating ill health”* [69].

There are multiple definitions of rural used in the academic literature and in policy arenas, we considered the term broadly based on population density and total population [14] and proximity to metropolitan zones (a measure of remoteness, which result in some small but remote cities being included) [55]. The search time frame was limited to the past 20 years in order to capture the most recent waves of immigrants who represent some of the most ethnically diverse and highly educated immigrant cohorts in Canada’s history [24]. Our study is focused on permanent settlement and health and therefore we excluded studies with temporary migrant workers. We also limited the language of publication to English. A broad but strategic search was conducted of literature in four major databases Web of Science, Scopus, PsycInfo, and PubMed. Our search algorithm included the following terms, synonyms and Boolean operators: [“rural” OR “small communit*” OR “small cit*” OR “remote” OR “countryside” OR “non-metropolitan” NOT “urban”] AND [“immigrant*” OR “newcomer*” OR “settle*” OR “immigration”] AND [“Canada”]. We included and excluded studies based on whether the studies directly discussed immigrant health or addressed a social determinant of health, including immigrant services, access to health services, housing, transportation, employment or social capital [69].

**Figure 1:**
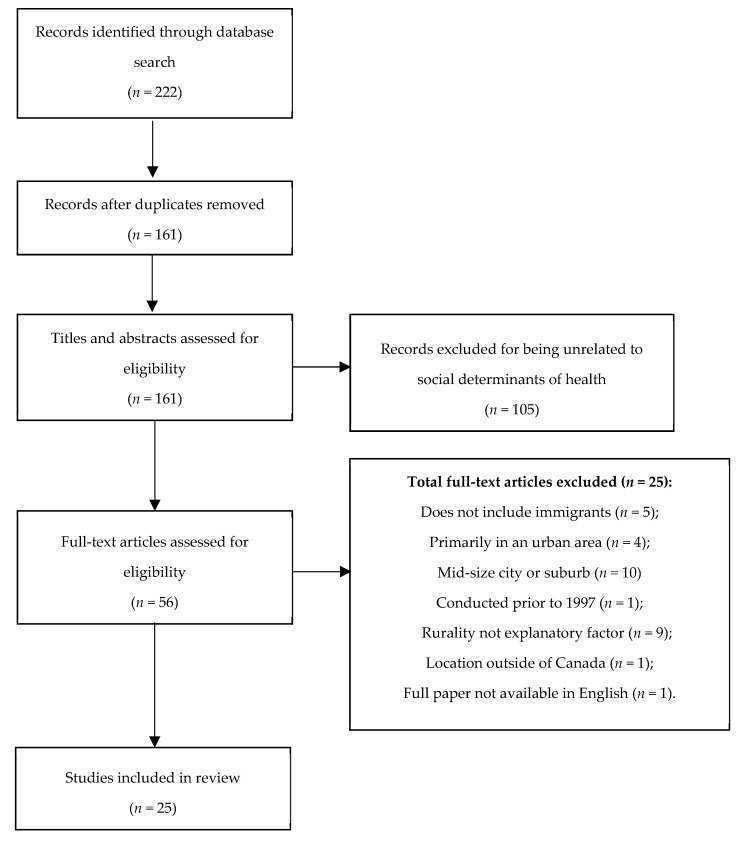
PRISMA chart for scoping review on immigrant settlement into rural communities and related determinants of health [70].

With respect to Step 2, included articles were those involving primary and secondary migration to rural areas, dealt with social determinants of health either directly or indirectly, and had explicit attention to rural communities (rather than a comparison between urban and rural). We depict our search outcome using a Preferred Reporting Items for Systematic Reviews and Meta-Analyses (PRISMA) flowchart (see Figure 1 below) [70].

Data were extracted from articles and summarized in a chart with categories (i.e., study purpose, research design, geographic location, population characteristics, social determinants of health, direct determinants of health, future research recommendations) that were agreed upon by the team (see Table A1 in Appendix). Once the data were extracted, a synthesis of the articles was conducted and significant areas of consensus, challenges, and gaps in knowledge were identified.

The final consultation step in our research involved interviewing 17 key informants across Canada on the topic of rural immigrant settlement and its potential intersectional impacts on the determinants of health, including how to facilitate healthy communities and future policy priorities. To gain information from multiple sectors of practice, our team sampled informants that had diverse expertise in the topic. Approximately nine out of 17 informants (53.0%) were researchers or private consultants, while four out of 17 informants (23.5%) were affiliated with a non-government organization, and another four out of 16 informants (23.5%) were government employees. Geographically speaking, participants were located in Manitoba (5/17) and Ontario (12/17). We used the consultations to contextualize the findings from the literature review.

## 3. Results

With respect to geographic location, 13 out of 25 studies were in rural communities, another 11 out of 25 studies were cross-sectional studies that compared rural and urban communities, and one out of the 25 studies was located in the small city of North Bay, Ontario. The most common research design found in the articles were qualitative studies (16/25), many of which were ethnographic in nature and included an interview component to gather immigrant experiences [71]. The remainder of the studies were quantitative (7/25) or mixed-methods studies (2/25).

With regards to the study populations, the majority of articles were focused on immigrants (22/25), whereas one of the 25 studies was focused on settlement service agencies, and another three out of 25 studies were based upon self-identified ethnic minorities, which included immigrants. While few (4/25) of the studies explicitly mentioned “social determinants of health”, our data extraction and analysis found five determinants were implicitly addressed in the literature; they are discussed in order of prominence here.

### 3.1. Determinant 1: Social Inclusion

The theme of social inclusion, cultural sensitivity, discrimination and social support-related topics are common (19/25) when exploring immigrant and minority experiences in rural communities [3,26,33,35,48,49,50,72,73,74,75,76,77,78,79,80,81,82,83]. Rural communities are found to be more socially ‘tight-knit’ due to a smaller population size [50,73]. Immigrants often perceive themselves as outsiders in a small community and report this dynamic to be a challenge to meaningful participation in community gatherings or other social events [73,74]. In discussion of the native versus non-native populations in rural communities, in the book titled *Eating Chinese: Culture on the Menu in Small Town Canada*, Lily Cho provided a narrative account of how Chinese food has been adapted in small towns throughout Canada [75]. A central theme in the book was the Chinese diaspora’s self-employment and agency in owning restaurants, and the recognition of how their experiences of food differ from their predominantly white customer base [75]. Resonating with the “other” dynamic found in Cho’s (2010) book, a small study of the experiences of ethnic minority students, including adolescent immigrants, that reside in southwestern Ontario, found that 15 out of 15 participants had experienced barriers to acceptance in the larger society [26]. The authors noted that the barriers may be the result of social and political structures (interethnic group dynamics) that perpetuate intolerance in the small community that they lived in, as residents express that immigrants are “taking up all the jobs” and “changing everything” [26]. In a case study of democratic citizenship education in a rural high school, there was a sentiment of intolerance among a small portion of students in the classroom, because they echoed the fear of immigrants stealing employment [48] (p. 180).

Additionally, social isolation for immigrants was found to be more prevalent in places that are not in proximity to the “prototypical” urban city [49]. For instance, in a national cross-sectional study using data from the Ethnic Diversity Survey comparing urban metropolitan and rural non-metropolitan settings, and visible minorities versus white (North American) and European groups, a larger percentage (between 67 to 75%) of visible minorities reported race as a cause for discrimination [49]. In the same study, the percentage of reported discrimination was lower in metropolitan centres in comparison to small communities [49].

Findings on social isolation among immigrant and minority ethnic groups vary from study to study. For instance, in contrast to the idea of immigrant isolation, a cross-sectional regional study based on the Canadian Community Health Survey found that there were no differences between immigrants and Canadian-born residents across Canada in terms of community belonging [3]. In an ethnographic study that was conducted in rural Florenceville–Bristol in the eastern province of New Brunswick, the author found that despite the rhetoric of fear of immigrants taking the jobs of local citizens, positive stories did shed light upon welcoming attitudes towards immigrant residents in the community [23]. In this study, the presence of self-identified ethnic minority adolescents, including new immigrant students incited broader acceptance with other members of the community and motivated citizens to positively engage with new residents [23].

The notion of “tight-knittedness” in rural communities for Canadian-born versus immigrant populations was noted as important for predicting social isolation [25] and for building social networks [49]. Further, the relationship between a lack of transportation availability and barriers to social integration in smaller communities is also noted [25,73,81,82,83]. As newcomers navigate employment and social interaction opportunities in small towns, transportation often plays a large role in determining their ability to meaningfully participate in community activities [72,73,82].

### 3.2. Determinant 2: Access to Culturally Appropriate Health and Settlement Services

A central topic (13/25) across studies was the various barriers that immigrants face accessing services that suit their settlement and healthcare needs. Key barriers include communication, transportation, financial constraints, time constraints, and overall lack of information [25,33,72,73,74,76,77,78,79,80,81,82,83,84]. Language or communication is a primary barrier for immigrants in their access to health, educational, and social services [25,73,76,77,83]. This finding is exemplified in a cross-sectional study of 212 newcomers in the Brant–Haldimand–Norfolk region, in which 54% of participants reported language as a major barrier in accessing physical or mental health services [82]. Other barriers to accessing services noted in the aforementioned study were employment status (57%), discrimination (46%) and stigma related to mental health (39%) [82].

Studies suggest that cultural differences between immigrants and Canadian-born people can be a significant barrier as well. Studies have found that there is a stigma attached to receiving mental health services for immigrants because one’s mental health is considered a private matter [81]. In a study of longitudinal trends in mental health between different ethnic groups across Canada, comparing urban and rural regions, it was found that immigrants’ overall health declined over time [80]. In this study, the authors suggested this may be due to the fact that some immigrants do not seek help for their mental health because of cultural barriers [80]. Similarly, in a study of belonging and mental health among the Indian diaspora population in rural Canada, it was found that language and cultural differences were barriers not only to integration and participation in the community, but as a result this also had a negative impact on individuals’ mental health [73].

In the policy areas of settlement and health services, studies conclude that there is a need for increased language support and training for newcomers to reduce communication barriers [74,85]. After studying the success of the Provincial Nominee Program in rural Steinbach, Manitoba, authors Carter et al. (2008) found that in order to foster successful integration of immigrants into a smaller community setting, it is important to have flexible delivery of services and programs [25]. For instance, having an English as a Second Language (ESL) course after work hours may encourage increased attendance and have a positive impact on immigrants who cannot attend ESL classes during the day [25].

The concept of equitable and culturally appropriate access to health care is also explored by an article that calls for dialogue among policymakers and health care providers to validate and better understand the meaning of health for minority women [83]. Women’s experiences of settlement are influenced not only by their newcomer status, but also by her domestic duties in a new environment [81]. To better understand how to improve health literacy among immigrant women, a study in Southwestern Ontario concluded that the utilization of participatory education can be beneficial [79]. In the aforementioned study, women were able to use metaphors to explain their experiences with moving from Mexico to a rural community in Southwestern Ontario, and the new culture that they have adapted when they arrived [79]. This narrative approach helped to explain experiences that were not easily translatable to health professionals. To address the unique health-based needs of women, their voices should be used in narrating settlement experiences [81]. Another recommendation is to focus on ‘women-centred’ approaches in healthcare through training programs for care-providing professionals [77].

### 3.3. Determinant 3: Gender

The intersectionality between gender, ethnicity, age, immigrant status, socio-economic status, and the experiences of health care in Canada was explored in multiple (11/25) studies [3,34,73,74,76,77,78,79,80,81,82,83]. In an ethnographic investigation of maternity healthcare experiences for 31 immigrant women who live in rural Alberta, it was found that participants’ cultural background and non-verbal communication, in addition to language barriers, lead to misunderstandings between physicians and women [76]. Another study found that minority-race immigrant women are less comfortable speaking to male health care professionals about sexual health in comparison to female practitioners [83]. These barriers are also found in a study that looked at the barriers and facilitators in family planning across Canada [78]. It was noted that there was a regional variation in family care, and particularly vulnerable populations included new immigrants, youth and women in rural communities [78]. A primary example of the intersectional nature of the gendered settlement experience is a woman who is migrating to Canada under the Family Class Sponsorship Policy, which renders her financially dependent on her spouse during the first few years of settlement, thereby imposing a further “marginalized status” in contrast to her financially independent spouse [81] (p. 68). To learn more about overall health for immigrant women, further research needs to be done to understand the intersectionality between gender, settlement and its effect on immigrant women’s health [81]. There also needs to be an assessment of cultural awareness among healthcare professionals [76].

### 3.4. Determinant 4: Employment

A common topic of discussion in reviewed articles is employment within rural communities (6/25). Due to the challenge of retaining employees in smaller regions across Canada, reviewed studies have focused on the role of foreign-trained professionals to fill gap in front-line health care workers. Including physicians [49]. In a policy brief on the ‘dilemma’ of physician shortage, it was found that foreign-trained professionals often work in rural areas until their contracts are over, and then move to an urban centre [86]. One study found that this gap may be filled by issuing provisional licenses to foreign physicians trying to gain the right to practice in Canada if they locate in rural or under-sourced areas as a prerequisite to gaining a full license [87]. The employment of foreign-trained professionals in rural communities has been distinctively analyzed to better understand policy options for managing migration from foreign countries [49,87]. For instance, a study of migration from sub-Saharan Africa to rural Canada found that despite the burden of disease and a shortage of physicians in Africa, health professionals pursue migration to Canada because of pull factors such as higher remuneration, safety, and a democratic political system [49]. Canada’s unique health care system and reliance upon foreign physicians was found to have a perverse effect upon source countries that critically need domestic health workers [49].

Challenges that persist in rural communities include economic stress and the ability to attract and retain newcomers [25,34,35,49]. In a mixed-methods study comparing pathways to employment between cities and rural communities, it was found that formal job-postings provided better access to jobs in rural regions for professionals [35]. Of note, however, in the aforementioned study it was also found that newcomers to the community that do not have pre-arranged employment have a more difficult time accessing social networks that can lead to employment [35]. The ratio difference between the successful use of weak social ties to find a job between immigrants (1.13) versus Canadian residents who have lived in Canada for over 30 years (4.80) was significant; thus, length of residency in Canada and one’s immigration status is a strong predictor of job-finding ability in rural communities [35]. Interestingly, while foreign professionals continue to fill in labour gaps, immigrants are paid less on average [84]. This is further magnified by the reduced social capital held by immigrants in rural areas, which can have an impact on both employment and income [35].

Further, immigrants were also found to be at greater risk for injury in the workplace, indicating a lack of knowledge and access to preventative occupational health and safety information [85]. Given the identifiable regional disparity between immigrant and non-immigrant income levels there is an opportunity to further research the persistence of income gaps [49,84] and the related disparity between these groups’ occupational health and safety risks [85].

### 3.5. Determinant 5: Housing

A prominent challenge noted in the literature (6/25) was related to securing affordable housing for rent and ownership [3,25,49,72,73,84]. In the small city of North Bay, Ontario, participants in a study exploring housing experiences indicated that there was a lack of availability of short-term yet affordable rental properties for newcomers [72]. Furthermore, newcomers were unaware of their rights as tenants residing in private property and were asked to do additional tasks that were not legally required [72]. This also has to do with the short timeframe between immigrants’ arrival in Canada and the need to secure housing [72]. Findings from the Ethnic Diversity Survey on economic and housing characteristics of ethno-cultural groups by location found that visible minorities in non-metropolitan centres were less likely to own a house at a rate of 78.9%, in comparison to European immigrants at 83.1% and white North American-born population at 79.4% [49]. This finding is particularly important for assessing immigrant health and settlement experiences because rural communities tend to have less or no resident-dense apartment complexes, in which people can rent housing. Additionally, this study found that housing can “shape” social relations and can have an impact on sentiments of discomfort and discrimination [49] (p. 242).

Studies have indicated that future research needs to examine the differences in housing experiences between immigrants in a small community versus a large city [3,72]. For the purpose of addressing equitable access to housing, Brown (2017) has recommended that municipal governments in small communities centralize publicly accessible housing information [72]. In addition, policymakers are encouraged to foster collaboration between government and housing service providers to address issues that immigrants have, including matters of affordability, availability and information about temporary housing options [72]. Similarly, Kitchen et al. (2012) recommend increasing the creation of affordable housing units through formal planning efforts [3]. Hall and Khan (2008) call for urban policy to alleviate the “sociospatial polarization” that underlys the regional disparities in housing and transportation opportunities [84]. Scholars have also echoed a call for more inclusive and multi-disciplinary policy-making that reflects the needs of vulnerable populations, including immigrants [25,72,82,83].

## 4. Discussion

This scoping review provided insight into the state of knowledge on the health impacts of the changing settlement patterns among Canada’s immigrant population. While this is an underexplored area of research with few studies directly linking rural settlement to health, our review found that there were five social determinants of health that shed light onto potential health implications for immigrants: social inclusion, culturally appropriate health and settlement services, gender, employment, and housing. While we attempted to categorize studies into one or more of these categories, often the determinants were interrelated (e.g., access to housing and employment influenced by social capital; access to health care services dependent on gender and intersectionality).

The determinant of greatest focus in the literature is social inclusion. This attention is well-deserved given the ethno-cultural, linguistic, and religious diversity of recent waves of immigrants in Canada compared to the relative social homogeneity in rural Canada [18], and the potential for negative encounters and feelings of isolation for immigrant minorities. While the majority of studies found social inclusion to be challenging in rural communities, other studies found that in rural communities a “tight-knit” community helped newcomers feel a sense of belonging, concluding that social inclusion is location-dependent. This is determined by existing social cohesion in rural communities [25,50] or by the implementation of new policy programs such as Welcoming Communities initiatives and Local-Community Integration Partnerships [34,37] that directly focus on community readiness for the arrival of diverse immigrant populations. The impacts of these policies on immigrants’ feelings of social inclusion and sense of belonging are an important direction for future research.

Access to health care and settlement services was widely discussed in the literature and directly connected to the health outcomes of immigrants. Rural areas are already recognized as underserved with respect to health care services [47], but this was exacerbated by the need for culturally appropriate care (e.g., language, cultural interpretations of health) among the diverse immigrant populations [82,83]. There is great potential for the targeted recruitment of foreign-trained health professionals to meet the health care needs of both the existing rural population and the growing immigrant populations in rural communities.

The literature pays particular attention to the experiences of immigrant women and the role of intersectionality in rural settlement. As a social determinant of health, gender identity shapes access to and experiences of other health determinants [33] including access to health and social services, social inclusion, and employment as noted in this review. Most relevant to the purpose of this review were the studies emphasizing the impact of rural settlement on mental health for female migrants who reported challenges with social isolation and redefined cultural norms [80,81,82]. There is a need to consider how to better support immigrant women as they transition between different cultures and geographies, particularly with respect to mental health. As Sethi (2013) has stated, “Unquestionably, individuals articulate their mental health in relation to their context, such as socially established roles and norms, religious beliefs, mind/body epistemology, and ontology” [83] (p. 528). Immigrants’ construction and experience of health is underexplored the literature in comparison to research that explores barriers to access.

With respect to the sparse research on rural employment of immigrants, this is a surprising gap in the literature given the prevalence of political discourse touting the economic benefits of immigrants in labour-strapped rural areas [1,33]. Indeed, the majority of literature focuses on temporary workers, but there is little investigation of skilled immigrants in rural areas beyond foreign trained doctors [49,86]. The implications for rural employment on health may prove to be positive, especially considering the widely noted barriers to employment that many immigrants face in urban areas, and the importance of employment for facilitating the settlement process [62]. This is an important area of research in Canada, particularly because of the investments in targeted immigration initiatives like the Provincial Nominee Program [30] and Ontario’s Rural Employment Initiative [29].

Finally, housing was a determinant of health noted in the literature though its prominence was small in comparison to the plethora of studies that focus on immigrant housing in more urban settings [88,89]. Overall, there is limited literature with regards to housing policy in rural communities and its ability to meet the needs of residents who require affordable housing for purchase or rent. Further, the need of larger immigrant families to secure suitable housing in rural areas has not yet been a focus of research in Canada, but if the settlement trends continue, this will be a significant issue for settlement and health. Evidence suggests that a rural community suited for all residents is one that has established inclusive policies in relation to transportation, housing, and social services [34,51]. Future research should focus on exploring levels of awareness and preparedness among rural planners, policy and decision-makers to determine the degree to which diversity and inclusion of immigrant populations is being factored into their decision-making [90].

This review has highlighted several gaps in understanding the health inequities for rural residing immigrants in Canada. First, there is a paucity of research examining the characteristics of a rural neighbourhood setting (e.g., housing configuration, active transportation opportunities) that can inhibit or enhance the settlement experiences and health of immigrants. Transportation was widely overlooked in the literature, though car dependency is often the only option in rural areas, a major contradiction to the widespread advocacy for more sustainable and active transportation in communities [46,51,91].

Second, there is a gap in understanding self-reported or objectively measured physical and mental health status of this population. The majority of studies gathered subjectively defined perceptions of health but no systematic study of self-reported or measured health status among rural residing immigrants exists. This is an important gap in knowledge preventing evaluation of health change over time, and the potential influence of rural settlement on the healthy immigrant effect.

Third, age is an underexplored and potentially beneficial avenue of understanding in relation to immigrants that reside in rural communities. Two in every five Canadian children under the age of 15 have an immigrant background [37]; however, none of the articles that were reviewed discussed the health implications of rural settlement for immigrant children. Only three out of the 25 articles reviewed looked at young adult or adolescent experiences of settlement or social integration into smaller communities [26,50,80]. Given the large demographic of young immigrants, further exploration of their experience is warranted; this is an under-explored avenue of academic literature and could be beneficial in understanding intersectional experiences for young minority immigrants that live in rural communities.

### Limitations

A primary limitation of scoping reviews is that they do not assess the quality or validity of the studies that are included [66]. Rather, in this study a descriptive approach to understanding the evidence was used and emphasized to synthesize data. A second limitation is that there is not a significant amount of existing data on the topic of rural immigrant settlement and health in Canada, thereby resulting in only 28 studies included. Given this paucity of data, we have cast a broad scope and categorized articles based on their discussion of the social determinants of health, even when authors did not acknowledge health as a direct impact of rural settlement. A third limitation is that this study has assessed Canadian literature and excluded articles on other countries such as the United States, Australia, and New Zealand. The rationale for this choice was based on the importance of considering local context when assessing place effects on health [38]. Canada’s new rural settlement policy initiatives, its adoption of the Welcoming Communities approach, and its prominent multiculturalism and diversity rhetoric all influence how and whether social determinants are/will be made accessible to immigrants settling in rural areas. While our review can be used to enhance a broader understanding of the potential pathways through which immigrant health is influenced by changing settlement patterns, there is a need to more directly assess health in Canada and abroad.

## 5. Conclusions

The international trend of immigrant settlement beyond cosmopolitan urban centres is expected to increase in the future as strategic immigration policy initiatives aim to off-set aging and declining populations in small towns and rural areas [30,92]. Given the existing health disparities for both immigrant populations and rural populations, this emerging settlement trend presents a potential double burden for health inequities among resettled immigrants in rural areas. This review of literature suggests that there is a significant gap in knowledge on how rural life impacts immigrant health. Existing research on the social determinants of health suggests that social inclusion, access to culturally appropriate health and social services, gender and intersectionality, employment, and housing are currently garnering the most attention by researchers, though the direct links to health are absent.

Through this review we have highlighted trends in social determinants of immigrant health in rural communities, though the mechanisms through which those determinants may help or hinder health status over the settlement trajectory is still unknown. For instance, is the reduced access to health care services offset by the employment opportunities present in rural areas? Do opportunities for more affordable housing (in comparison to highly urbanized areas) mitigate the loss of social networks? An assessment of the impact of these competing health determinants on self-reported health and diagnosed health conditions is necessary to understand the impact of changing settlement patterns on the healthy immigrant effect and health disparities in the immigrant population. The scarcity of data indicates a timely opportunity to better understand and facilitate healthy, diverse communities throughout rural Canada and abroad.

## Figures and Tables

**Table 1:** Applying the stages of scoping review protocol [67].

Review Step	Application
Step 1: Identifying research question	The research question guiding this study is: “How do rural settlement patterns influence health and social determinants of health for immigrant populations in Canada?”
Step 2: Identifying relevant studies	The following data sources were to be used for this study: PsycInfo, PubMed, Web of Science, and Scopus. Inclusion criteria Population: Immigrants, foreign trained professionals, newcomers Literature: English Location: Rural communities in Canada Exclusion criteria Located in an urban area and does not compare to rural area Located in a mid-size city or suburb Located outside of Canada Does not include immigrants Rurality not an explanatory factor for health status Conducted prior to 1997 Paper not available in English
Step 3: Selecting studies	The team used an iterative approach to eliminate studies based on exclusion criteria and contents that do not address the research question or fulfill our objectives.
Step 4: Charting data	A descriptive summary table was used to highlight characteristics found in articles (refer to Table A1 in Appendix). A common framework was used to collect data from all articles to track themes found in the data.
Step 5: Summarizing and reporting the results	This article summarizes the results of this study and provides discussion of existing literature and its effect on future research on this topic.
Step 6: Consultation	Approximately 17 key informants were interviewed across Canada on this topic from multiple sectors of practice. Findings from the consultation will be elaborated in a forthcoming article.

## Appendix A

**Table A1:** Summary of included articles.

Title	Author(s)	Year	Location Type	Literature Type & Purpose	Theme(s)
Housing Experiences of Recent Immigrants to Canada’s Small Cities: the Case of North Bay, Ontario	Brown N.R.	2017	Small city	Ethnographic study: To better understand newcomers’ ability to attain affordable housing in North Bay	Social inclusion Barriers to access Housing affordability
Attracting immigrants to smaller urban and rural communities: Lessons learned from the Manitoba Provincial Nominee Program	Carter, T., Morrish, M., & Amoyaw, B.	2008	Rural communities	Case study: To better understand the changing distribution and settlement of immigrants	Barriers to access Rural employment Housing affordability
Belonging and Mental Wellbeing Among a Rural Indian-Canadian Diaspora: Navigating Tensions in “Finding a Space of Our Own”	Caxaj, C. Susana; Gill, Navjot K.	2017	Rural community	Ethnographic study: To explore sense of belonging in a rural community	Social inclusion Barriers to health access Gendered experiences
Examining the relationship between social support availability, urban center size, and self-perceived mental health of recent immigrants to Canada: A mixed-methods analysis	Chadwick K.A., Collins P.A.	2015	Rural vs. urban communities	Mixed-methods study: To explore the relationship between immigrants’ self-perceived mental health and urban center size	Social inclusion Barriers to access Gendered experiences Housing affordability
Eating Chinese: Culture on the menu in small town Canada	Cho, L.	2010	Rural community	Narrative: To discuss the Chinese diaspora in rural communities across Canada and understand how their cuisine and entrepreneurship have evolved over time	Social inclusion
Differences in hi-tech immigrant earnings and wages across Canadian cities	Hall, P. V., & Khan, A. J.	2008	Rural vs. urban communities	Cross-sectional study: To compare immigrants vs. non-immigrants’ wages across different geographies	Barriers to access Rural employment Housing affordability
Leading a diverse school during times of demographic change in rural Canada: Reflection, action and suggestions for practice	Hamm L., Doğurga S.-L., Scott A.	2016	Rural community	Literature review: To analyze teaching practices in relation to demographic change in rural Canada	Social inclusion Barriers to access
An ethnographic study of communication challenges in maternity care for immigrant women in rural Alberta	Higginbottom G.M.A., Safipour J., Yohani S., O’Brien B., Mumtaz Z., Paton P.	2015	Rural community	Ethnographic study: To better understand communication challenges for immigrant women in seeking maternal healthcare in rural Alberta, Canada.	Social inclusion Barriers to access Gendered experiences
An ethnographic investigation of the maternity healthcare experience of immigrants in rural and urban Alberta, Canada	Higginbottom G.M., Safipour J., Yohani S., O’Brien B., Mumtaz Z., Paton P., Chiu Y., Barolia R.	2016	Rural vs. urban communities	Ethnographic study: To better understand and compare maternity healthcare for immigrant women in rural and urban Canada	Social inclusion Barriers to access Gendered experiences
Barriers and facilitators to family planning access in Canada	Hulme J., Dunn S., Guilbert E., Soon J., Norman W.	2015	Rural communities	Ethnographic study: To explore barriers to family planning in Canada for vulnerable populations in rural areas	Social inclusion Barriers to access Gendered experiences
The dilemma of physician shortage and international recruitment in Canada	Islam, N.	2014	Rural communities	Policy brief: To summarize and analyze policies that relate to foreign-trained physicians (rural and urban).	Rural employment
Sense of community belonging and health in Canada: A regional analysis	Kitchen, P.; Williams, A.; Chowhan, J.	2012	Rural vs. urban communities	Cross-sectional study: To look at self-perceived mental health and community belonging across different geographic contexts (urban vs. rural) and different groups of people	Social inclusion Gendered experiences Housing affordability
Managing health professional migration from sub-Saharan Africa to Canada: a stakeholder inquiry into policy options	Labonté, R., Packer, C., & Klassen, N.	2006	Rural vs. urban communities	Ethnographic study: To better understand the migration of foreign-trained health professionals and its implications for Canada (rural to urban transect).	Rural employment
The Power of Collaborative Inquiry and Metaphor in Meeting the Health Literacy Needs of Rural Immigrant Women	Lauzon, A.; Farabakhsh, R.	2014	Rural community	Ethnographic study: To use a participatory method to understand needs of immigrant women living in a rural Ontarian community	Social inclusion Gendered experiences
Social capital, labour markets, and job-finding in urban and rural regions: Comparing paths to employment in prosperous cities and stressed rural communities in Canada	Matthews R., Pendakur R., Young N.	2009	Rural vs. urban communities	Mixed-methods study: To compare social capital utilization and employment opportunities between rural and non-rural settings	Social inclusion Rural employment
The migration decisions of physicians in Canada: the roles of immigrant status and spousal characteristics	McDonald J.T., Worswick C.	2012	Rural vs. urban communities	Cross-sectional study: To study mobility patterns of immigrants and non-immigrant physicians (to rural areas).	Rural employment
Longitudinal trends in mental health among ethnic groups in Canada	Pahwa P., Karunanayake C.P., McCrosky J., Thorpe L.	2012	Rural vs. urban communities	Longitudinal study: To examine longitudinal trends between mental health and other demographic factors within ethnic groups	Social inclusion Barriers to access Gendered experiences
Democratic citizenship education in rural high schools: Navigating difference and conflict in three Ontario classrooms	Pattison-Meek, J. M.	2016	Rural communities	Ethnographic study: To analyze and compare democratic participation in rural and urban high schools	Social inclusion
Experiences of discrimination and discomfort: A comparison of metropolitan and non-metropolitan locations	Ray, B.; Preston, V.	2013	Rural vs. urban communities	Cross-sectional study: To examine discrimination among visible vs. non-visible minority groups across varying geographic settings	Social inclusion Housing affordability
Intersectional exposures: Exploring the health effect of Employment with KAAJAL immigrant/refugee women in Grand Erie through Photovoice	Sethi B.	2013a	Rural vs. urban communities	Cross-sectional study: To survey immigrants’ perception of challenges in relation to health services	Social inclusion Barriers to access Gendered experiences
Newcomers health in Brantford and the counties of Brant, Haldimand and Norfolk: Perspectives of newcomers and service providers	Sethi B.	2013b	Rural communities	Literature review: To demonstrate policy influences over immigrant women’s’ access to health services	Social inclusion Barriers to health access Gendered experiences
Service delivery on rusty health care wheels: Implications for visible minority women	Sethi, B.	2014	Rural/small community	Ethnographic study: To use photovoice to narrate immigrant women’s experiences in relation to employment	Social inclusion Barriers to access Gendered experiences
The unequal distribution of occupational health and safety risks among immigrants to Canada compared to Canadian-born labour market participants: 1993–2005	Smith P.M., Mustard C.A.	2009	Rural vs. urban communities	Cross-sectional study: To compare and examine health and safety risks among immigrants vs. non-immigrants	Barriers to access
Understanding the leisure experiences of a minority ethnic group: South Asian teens and young adults in Canada	Tirone, S; Pedlar, A	2000	Rural communities	Ethnographic study: To study the effects of leisure activity among ethnic minority students in a small town	Social inclusion
Getting Used to the Quiet: Immigrant Adolescents’ Journey to Belonging in New Brunswick, Canada	Wilson-Forsberg S.	2012	Rural communities	Ethnographic study: To examine the inclusion of immigrant adolescents into a small community in New Brunswick, Canada	Social inclusion
